# Mitochondrial fission factor (MFF) frameshift variant in Bullmastiffs with mitochondrial fission encephalopathy

**DOI:** 10.1111/age.13263

**Published:** 2022-09-09

**Authors:** Matthias Christen, Rodrigo Gutierrez‐Quintana, Helene Vandenberghe, Adriana Kaczmarska, Jacques Penderis, Roberto José‐López, Angie Rupp, Ian R. Griffiths, Vidhya Jagannathan, Tosso Leeb

**Affiliations:** ^1^ Institute of Genetics, Vetsuisse Faculty University of Bern Bern Switzerland; ^2^ School of Veterinary Medicine, College of Medical Veterinary and Life Sciences University of Glasgow Glasgow UK; ^3^ Highcroft Veterinary Referrals Bristol UK; ^4^ Vet‐Extra Neurology Stirling UK; ^5^ Hamilton Specialist Referrals IVC Evidensia High Wycombe UK

**Keywords:** animal model, *Canis lupus familiaris*, dog, mitochondrion, neurology, precision medicine, veterinary medicine

## Abstract

Familial cerebellar ataxia with hydrocephalus in Bullmastiffs was described almost 40 years ago as a monogenic autosomal recessive trait. We investigated two young Bullmastiffs showing similar clinical signs. They developed progressive gait and behavioural abnormalities with an onset at around 6 months of age. Neurological assessment was consistent with a multifocal brain disease. Magnetic resonance imaging of the brain showed intra‐axial bilateral symmetrical focal lesions localised to the cerebellar nuclei. Based on the juvenile age, nature of neurological deficits and imaging findings, an inherited disorder of the brain was suspected. We sequenced the genome of one affected Bullmastiff. The data were compared with 782 control genomes of dogs from diverse breeds. This search revealed a private homozygous frameshift variant in the *MFF* gene in the affected dog, XM_038574000.1:c.471_475delinsCGCTCT, that is predicted to truncate 55% of the wild type MFF open reading frame, XP_038429928.1: p.(Glu158Alafs*14). Human patients with pathogenic *MFF* variants suffer from ‘encephalopathy due to defective mitochondrial and peroxisomal fission 2’. Archived samples from two additional affected Bullmastiffs related to the originally described cases were obtained. Genotypes in a cohort of four affected and 70 unaffected Bullmastiffs showed perfect segregation with the disease phenotype. The available data together with information from previous disease reports allow classification of the investigated *MFF* frameshift variant as pathogenic and probably causative defect of the observed neurological phenotype. In analogy to the human phenotype, we propose to rename this disease ‘mitochondrial fission encephalopathy (MFE)’.

## INTRODUCTION

Familial cerebellar ataxia with hydrocephalus in Bullmastiff dogs was initially described in 1983 (OMIA 002551‐9615; Carmichael et al., 1983). Affected puppies of one litter showed ataxia, impaired vision and behavioural abnormalities consisting of hysterical behaviour, difficulty to train, backing compulsively when called and lifting a foreleg while eating. The clinical signs were macroscopically associated with symmetrical hydrocephalus and histologically with cerebellar vacuolisation, gliosis and axonal degeneration. It was hypothesised that the observed phenotype was due to a genetically determined metabolic disturbance with an autosomal recessive mode of inheritance (Carmichael et al., 1983). Four years later, the same author described a total of six closely related Bullmastiff dogs with similar clinical signs (Carmichael, 1987). Additionally, abnormally shaped mitochondria in axons of affected brain regions were found in electron micrographs, leading to the conclusion that the dogs suffered from a neuronal abiotrophy caused by an intrinsic metabolic effect (Carmichael, 1987). Magnetic resonance imaging (MRI) demonstrated changes within the deep cerebellar nuclei in additional affected dogs (Johnson et al., 2001).

The current study was prompted by reports of two cases of Bullmastiffs from different litters showing behavioural and gait abnormalities. We hypothesised that the dogs might be affected by the same disease that was first described almost 40 years ago and initiated a genetic investigation.

## MATERIALS AND METHODS

### Clinical examinations

An 8‐month‐old, female entire Bullmastiff (case no. 1) and a 6‐month‐old, male entire Bullmastiff (case no. 2) underwent physical and neurological examinations. Additional laboratory investigations included A complete blood cell count, serum biochemistry (including electrolyte levels) and serology for canine distemper virus (case no. 2 only), *Toxoplasma gondii* and *Neospora caninum*. Additionally, a cisternal cerebrospinal fluid sample (case no. 2) was submitted for protein levels and total and differential cell counts, and for this dog also urine was submitted to an external human testing laboratory for organic acid analysis.

### Magnetic resonance imaging examination

Magnetic resonance imaging of the brain was performed on case no. 2 with a 1.5 Tesla machine (1.5T Magnetom, Siemens) and included *T*_2_‐weighted sagittal, dorsal and transverse views, with the following transverse views: fluid attenuated inversion recovery and gradient echo *T*_2_*‐, *T*_1_‐weighted pre‐ and post‐contrast sequences (gadopentate dimeglumine; Magnevist, Bayer Schering Pharma AG).

### Post‐mortem examination

Owing to clinical progression, case no. 2 was euthanised and a post‐mortem examination was performed. Representative samples of heart, lungs, liver and brain were stained with hematoxylin and eosin and examined histologically.

### Genetic analyses

#### Animal samples

The study included samples from 74 Bullmastiffs. We used EDTA blood samples from the two contemporary clinical cases (case nos 1 and 2) and formalin‐fixed paraffin‐embedded (FFPE) tissue samples from two additional affected dogs seen in 1996 (case nos 3 and 4) that were reportedly siblings and closely related to the previously reported cases (Carmichael, 1987). The FFPE samples of the historical cases were kindly provided by the pathology service of the School of Veterinary Medicine of the University of Glasgow. EDTA blood samples of 70 additional Bullmastiffs that had been donated to the Vetsuisse Biobank were used as controls. They consisted of five unaffected close relatives of case no. 2 and 65 population controls without known relationships to any of the cases.

#### DNA extraction

Genomic DNA was isolated from EDTA blood with the Maxwell RSC Whole Blood Kit using a Maxwell RSC instrument (Promega). The same instrument was used for DNA isolation from FFPE tissue samples with the Maxwell RSC DNA FFPE kit.

#### Whole‐genome sequencing

An Illumina TruSeq PCR‐free DNA library with a ~390 bp insert size of case no. 1 was prepared. We collected 241 million 2 × 150 bp paired‐end reads on a NovaSeq 6000 instrument (27.5 × coverage). Mapping and alignment were performed as described in Jagannathan et al. (2019). The sequence data were deposited under the study accession no. PRJEB16012 and sample accession no. SAMEA8157163 at the European Nucleotide Archive.

#### Variant calling and filtering

Variant calling was performed using GATK HaplotypeCaller (McKenna et al., 2010) in gVCF mode as described by Jagannathan et al. (2019). To predict the functional effects of the called variants, snpeff software (Cingolani et al., 2012) together with NCBI annotation release 106 for the UU_Cfam_GSD_1.0 genome reference assembly was used. For variant filtering, we used 782 genetically diverse control dog genomes representing 175 different dog breeds and 21 mixed‐breed dogs (Table S1).

Private variants in the case were identified by a hard filtering approach of the vcf‐file using a Python script. Private variants were required to have a homozygous alternate genotype in the case (1/1) and a homozygous reference (0/0) or missing genotype (./.) in the controls. The control cohort did not contain any Bullmastiffs; therefore the probability of having a heterozygous carrier in the control group was estimated as negligible.

Variants with SnpEff‐predicted ‘high’ or ‘moderate’ impact were classified as protein‐changing variants. These include missense, nonsense, frameshift and splice site variants.

#### Gene analysis

We used the UU_Cfam_GSD_1.0 dog reference genome assembly and NCBI annotation release 106. Numbering within the canine *MFF* gene corresponds to the NCBI RefSeq accession nos XM_038574000.1 (mRNA) and XP_038429928.1 (protein).

#### PCR and sanger sequencing

The candidate variant *MFF*:c.471_475delinsCGCTCT was genotyped by direct Sanger sequencing of PCR amplicons. A 380 bp (or 381 bp in case of the mutant allele) PCR product was amplified from genomic DNA using AmpliTaqGold360Mastermix (Thermo Fisher Scientific) and the primers 5′‐CTCCCTTTCTTTGTGCCTCA‐3′ and 5′‐CGAGAGGATAATGCTACTGGAAA‐3′. A smaller PCR product of 101 bp size (or 102 bp in case of the mutant allele) was amplified from FFPE‐derived DNA with the primers 5′‐TCCCTTTCTTTGTGCCTCAC‐3′ and 5′‐CTCTGACCAGCTGTCCGTTT‐3′. After treatment with exonuclease I and alkaline phosphatase, amplicons were sequenced on an ABI 3730 DNA Analyzer (Thermo Fisher Scientific). Sanger sequences were analysed using the sequencher 5.1 software (GeneCodes).

## RESULTS

### Clinical investigations

An 8‐month‐old, female entire (case no. 1) and a 6‐month‐old, male entire (case no. 2) Bullmastiff presented with a chronic and progressive history of uncoordinated gait and abnormal behaviour including barking at imaginary objects and no interaction with other dogs, and decreased vision. The clinical signs were noticed by the owners a couple of months before presentation and had been gradually progressing. Some of the littermates were reported to be showing similar clinical signs. Physical examination was mainly unremarkable, apart from case no. 1 exhibiting scuffing wounds on the dorsal aspect of both pelvic limbs. Neurological examination showed abnormal mentation and a wide‐base stance (Video S1). The gait was abnormal with hypermetria affecting all limbs. Menace response was absent or decreased with normal pupillary light reflexes in both cases. Additionally, spontaneous and intermittent horizontal nystagmus was observed in case no. 2. Postural reactions (mainly hopping responses) were delayed in all limbs. No pain was elicited on spinal palpation and segmental spinal reflexes were normal. The neurological examination was consistent with a disease involving the cerebral cortex, leading to an abnormal behaviour and decreased vision, and the vestibulo‐cerebellum, leading to cerebellar ataxia and nystagmus.

**FIGURE 1:**
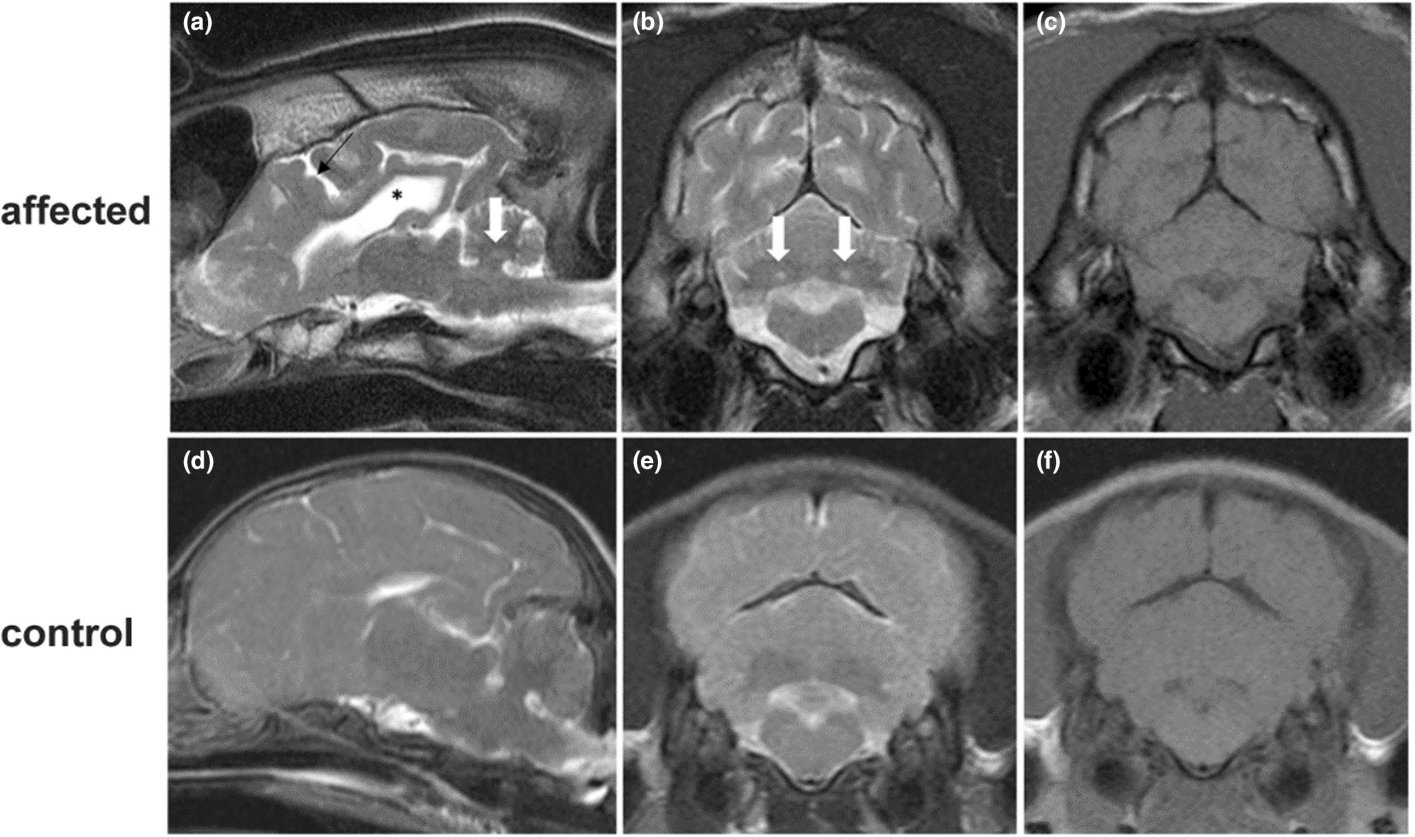
Magnetic resonance imaging of case no. 2 and an age‐matched control. (a) *T*_2_‐weighted parasagittal image of the brain showing widening of the cerebral sulci (black arrow), lateral ventricle enlargement (black asterisk) and hyperintensity of the cerebellar nuclei (white arrow). Transverse *T2*‐weighted image (T2WI) (b) and *T1*‐weighted image (T1WI) (c) at the level of the cerebellum showing bilateral and symmetric T2WI intra‐axial hyperintensities at the level of the cerebellar nulcei (white arrows); these lesions are isointense on T1WI (c). (d) *T*_2_‐weighted parasagittal of an age‐matched control with no widening of the cerebral sulci or enlargement of the lateral ventricle for comparison. Transverse T2WI (e) and T1WI (f) at the level of the cerebellum of an age‐matched control with no lesions.

Complete blood cell count and serum biochemistry including electrolyte levels showed a mild increase in cholesterol and creatinine kinase in case no. 2. Serologies for canine distemper virus, *Toxoplasma gondii* and *Neospora caninum* were negative. Cerebrospinal fluid and urine organic acids analyses for case no. 2 were within normal range. MRI of the brain showed intra‐axial bilateral symmetrical focal *T*_2_‐weighted hyperintense, *T*_1_‐weighted isointense and no contrast‐enhancing lesions localised to the cerebellar nuclei. Additionally, the sulci of the cerebral cortex appeared widened with an increased amount of cerebrospinal fluid and the lateral ventricles were bilaterally enlarged (Figure 1).

Case no. 1 died owing to progression of clinical signs. No further information regarding the age of the dog and circumstances of death are available. Case no. 2 was euthanised at 10 months of age owing to progression of the clinical signs. Post‐mortem examination confirmed spongy vacuolar changes in the cerebellar nuclei as previously reported (Carmichael et al., 1983). Mild to moderate dilation of the left cardiac ventricle, less so the right ventricle, compatible with a dilated cardiomyopathy, was observed, in turn considered the cause for the mild hydrothorax, ascites and hydropericardium present, as well as pulmonary congestion, hepatic centrilobular fibrosis and mild tunica media hyperplasia of the small arteries in the septal myocardium.

### Genetic analysis

At the beginning of the genetic analysis, we only had access to a single affected dog (case no. 1). As the clinical and neurological findings of this case resembled previously described cases in Bullmastiffs, we hypothesised that that the phenotype in the affected dog was due to a genetic defect with a monogenic autosomal recessive mode of inheritance. We sequenced the genome of case no. 1 and identified private homozygous variants that were not present in the genome sequences of 782 control dogs of diverse breeds (Table 1 and Tables S1 and S2).

**TABLE 1:** Homozygous variants in case no. 1, filtered against 782 control genomes

Filtering step	Variants
All variants in the affected dog	2 859 546
Private variants	591
Protein‐changing private variants	8

The resulting variants were prioritised according to functional knowledge of the affected genes. The automated bioinformatic analysis identified two independent homozygous private protein‐changing variants at neighbouring nucleotides in the functional candidate gene *MFF* encoding the mitochondrial fission factor. Visual inspection of the short read alignments in the affected region revealed that the two initially separately called variants actually represented just one single insertion–deletion variant. This variant, XM_038574000.1:c.471_475delinsCGCTCT, leads to a frameshift and is predicted to truncate 209 codons or roughly 55% of the wild type MFF open reading frame, XP_038429928.1:p.(Glu158Alafs*14). On the genomic level, the variant can be described as Chr25:40,322,999_40,323,003delinsCGCTCT (UU_Cfam_GSD_1.0 assembly). The other six private protein‐changing variants were not located in genes known to cause similar phenotypes in humans, mice or domestic animals.

**FIGURE 2:**
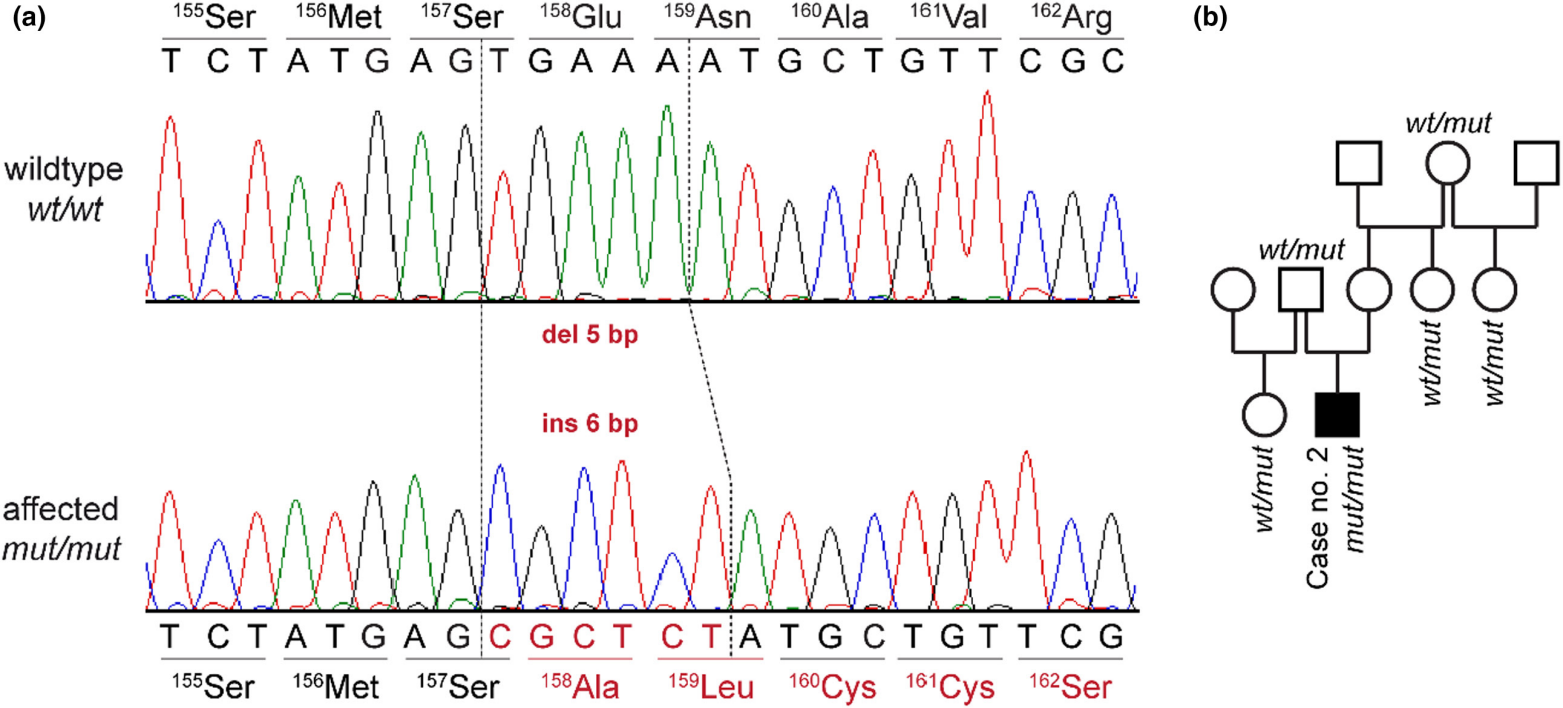
Details of the *MFF*:C.471_475delinsCGCTCT variant. (a) Representative electropherograms of a control and an affected dog are shown. The amino acid translations of the wild type and mutant alleles are indicated. (b) Pedigree of case no. 2. The genotypes at the *MFF*:C.471_475delinsCGCTCT variant are indicated for sampled dogs. The sire of case no. 2 was confirmed to be heterozygous as expected for an obligate carrier of a monogenic autosomal recessive trait.

We confirmed the presence of the *MFF* variant in a homozygous state in case no. 1 by Sanger sequencing (Figure 2a). At this point of the genetic analysis, we became aware of the second case with a very similar phenotype to the index case. We also genotyped the second case and five of its unaffected close relatives (Figure 2b).

Furthermore, we identified FFPE archived samples from two additional young Bullmastiff siblings seen in 1996 (cases no. 3 and 4) with similar clinical presentation and closely related to previously reported cases (Carmichael, 1987). We genotyped the *MFF* variant in the additional cases and a cohort of unrelated control Bullmastiffs (Table 2). These analyses confirmed a perfect genotype–phenotype association. All four available cases were homozygous mutant, one obligate carrier was heterozygous and none of the 70 unaffected dogs were homozygous for the mutant allele. The carrier frequency in the unrelated control cohort was 12/65 (18%).

**TABLE 2:** Association of the genotypes at the *MFF*:C.471_475delinsCGCTCT variant with ataxia and hydrocephalus in 74 bullmastiffs

Phenotype	Wt/Wt	Wt/Mut	Mut/Mut
Affected Bullmastiffs (*n* = 4)	–	–	4
Non‐affected relatives of case no. 2 (*n* = 5)	–	5	–
Unrelated control Bullmastiffs (*n* = 65)	53	12	–

## DISCUSSION

We describe two contemporary cases of Bullmastiffs with progressive behavioural and gait abnormalities exhibiting a similar clinical phenotype to dogs reported almost 40 years ago (Carmichael, 1987; Carmichael et al., 1983).

In this study, we provide a plausible candidate disease causing variant, *MFF*:c.471_475delinsCGCTCT. The *MFF* gene encodes the mitochondrial fission factor (Gandre‐Babbe & van der Bliek, 2008). Mitochondria are subject to a constant series of fusion and fission in order to regulate subcellular processes and their own homeostasis. Thus, mitochondrial morphology is dynamically regulated at all times (Meyer et al., 2017).

Loss of function variants in the *MFF* gene result in a cellular phenotype with elongated tubular mitochondria because the organelles can still fuse while fission is blocked (Gandre‐Babbe & van der Bliek, 2008; Otera et al., 2010). The resulting hyperfused mitochondria may be resistant to autophagosomal degradation leading to the accumulation of damaged and abnormally functioning mitochondria as well as reactive oxygen species (Meyer & Bess, 2012; Twig et al., 2008).

These mitochondrial defects result in a specific clinical phenotype in human patients with *MFF* variants, which is termed ‘encephalopathy due to defective mitochondrial and peroxisomal fission 2’ (OMIM# 617086, Shamseldin et al., 2012; Koch et al., 2016; Nasca et al., 2018; Panda et al., 2020). Changes to mitochondrial morphology with those exhibiting elongated tubular shapes have been observed both in human patients (Koch et al., 2016) and in affected dogs (Carmichael, 1987). Furthermore, bilateral symmetrical MRI changes affecting the basal and cerebellar nuclei have been reported in humans with *MFF* variants (Nasca et al., 2018) sharing some similarities with the MRI changes reported in the present and previously reported dogs (Johnson et al., 2001). Further studies will be necessary to understand the predilection for specific nuclei which appears to vary between species and/or variants.

*Mff* ^*−/−*^ knockout mice die prematurely with a mean life span of 13 weeks. The murine phenotype is characterised by a severe dilated cardiomyopathy in combination with prominent neuromuscular defects (Chen et al., 2015). Interestingly, the post‐mortem examination of one of the affected dogs showed mild to moderate dilated cardiomyopathy. Further studies will be needed to determine the degree and significance of the cardiac changes observed in dogs with this *MFF* variant.

The canine *MFF*:c.471_475delinsCGCTCT variant is predicted to truncate 55% of the open reading frame of the wild type *MFF* transcript, XP_038429928.1:p.(Glu158Alafs*14). We assume that the premature stop codon results in a complete loss of function. The mutant allele was absent in whole genome sequence data of 782 control dogs from genetically diverse breeds. Three additional affected Bullmastiffs with strikingly similar phenotypes carried the same homozygous mutant genotype, while 70 non‐affected Bullmastiff dogs were found to be free of the homozygous mutant genotype. The pedigrees of the affected dogs strongly suggested monogenic autosomal recessive inheritance (Carmichael, 1987). The phenotype with documented changes in mitochondrial morphology (Carmichael, 1987) is highly specific. Extrapolating the established guidelines for the interpretation of sequence variants in human medicine (Richards et al., 2015) to dogs, these arguments allow the *MFF*:c.471_475delinsCGCTCT variant to be classified as pathogenic.

The clinical and pathological features of this disease that closely resemble the human phenotype, together with the genetic findings, suggest MFF:c.471_475delinsCGCTCT as compelling causative variant. To the best of our knowledge, the affected dogs represent the first domestic animals described with an *MFF*‐related disease. In analogy to the human phenotype, we tentatively propose to rename the phenotype seen in Bullmastiffs ‘mitochondrial fission encephalopathy (MFE)’. Our results enable genetic testing, which can be used to avoid the unintentional breeding of further MFE‐affected dogs. In addition, the studied dogs might serve as a spontaneous large animal model to further the understanding of mitochondrial fission dysfunction in human patients.

## FUNDING INFORMATION

This research did not receive any specific grant from funding agencies in the public, commercial, or not‐for‐profit sectors.

## CONFLICT OF INTEREST

The authors declare no conflict of interest.

## ETHICAL APPROVAL

All animal experiments were performed according to local regulations. The dogs in this study are privately owned and were examined with the consent of the owners. The Cantonal Committee for Animal Experiments approved the collection of blood samples from control dogs that were used in this study (Canton of Bern; permit BE 71/19).

## Supporting information


Table S1

Table S2

Vedio S1

## Data Availability

The accession nos for the sequence data reported in this study are listed in Table S1.
